# Molecular and functional characterization of porcine poly C binding protein 1 (PCBP1)

**DOI:** 10.1186/s12917-023-03861-4

**Published:** 2024-01-13

**Authors:** Yue Song, Linqing Wang, Menglong Xu, Xiuxiang Lu, Yumin Wang, Limeng Zhang

**Affiliations:** 1 Molecule Biology Laboratory of Zhengzhou Normal University, Zhengzhou Henan, 450044 China; 2 College of Veterinary Medicine, Henan Agricultural University, Zhengzhou, Henan 450046 China

**Keywords:** Porcine poly C binding protein 1, Subcellular, Apoptosis, Cell cycles

## Abstract

**Background:**

Poly C Binding Protein 1 (PCBP1) belongs to the heterogeneous nuclear ribonucleoprotein family. It is a multifunctional protein that participates in several functional circuits and plays a variety of roles in cellular processes. Although PCBP1 has been identified in several mammals, its function in porcine was unclear.

**Results:**

In this study, we cloned the gene of porcine PCBP1 and analyzed its evolutionary relationships among different species. We found porcine PCBP1 protein sequence was similar to that of other animals. The subcellular localization of PCBP1 in porcine kidney cells 15 (PK-15) cells was analyzed by immunofluorescence assay (IFA) and revealed that PCBP1 was mainly localized to the nucleus. Reverse transcription-quantitative PCR (RT-qPCR) was used to compare PCBP1 mRNA levels in different tissues of 30-day-old pigs. Results indicated that PCBP1 was expressed in various tissues and was most abundant in the liver. Finally, the effects of PCBP1 on cell cycle and apoptosis were investigated following its overexpression or knockdown in PK-15 cells. The findings demonstrated that PCBP1 knockdown arrested cell cycle in G0/G1 phase, and enhanced cell apoptosis.

**Conclusions:**

Porcine PCBP1 is a highly conserved protein, plays an important role in determining cell fate, and its functions need further study.

**Supplementary Information:**

The online version contains supplementary material available at 10.1186/s12917-023-03861-4.

## Background

Poly C binding protein (PCBP) generally refers to an RNA binding protein belongs to the heterogeneous nuclear ribonucleoprotein family with a molecular weight of about 38 kDa [1]. There are four members in PCBP family, PCBP1–4, also known as α-CPs or hnRNP E 1–4 [2]. Three highly conserved hnRNP K homology (KH) domains and poly(C)-binding specificity are common features of the PCBP family [2, 3]. The KH domain is critical for PCBP1 to recognize and bind poly(C) DNA and RNA sequences in mammalian cells [4–6].

PCBP1 is highly expressed in a variety of human tissues and organs [7]. It is mainly located in the nucleus and distributed in the cytoplasm. A variety of biological processes, such as transcription, translation, protein interaction, and shuttling of mRNA between the nucleus and cytosol, are contributed by PCBP1 [8–12]. In addition, PCBP1 is involved in cell cycle regulation [13], and loss of PCBP1 can arrest cells in the G1 phase [14, 15]. PCBP1 can induce apoptosis, and Shi et al. confirmed that PCBP1 increased p27 expression by stabilizing p27 mRNA, further promoting apoptosis [16]. PCBP1 is involved in autophagy, and its overexpression can weaken the stability of microtubule-associated protein light chain 3 (LC3 B) mRNA, inhibit LC3 B expression, and lead to autophagy inhibition [17].

Pigs are an important species in the animal breeding industry, and virus invasion is one of the key issues for animal health and business development. In recent years, a variety of emerging and re-emerging infectious diseases have jeopardized the healthy development of the animal breeding industry. The development of targeted therapeutic drugs to improve porcine autoimmunity has become one of the methods to solve this problem. Innate immunity is the body’s first line of defense against infection by foreign pathogenic microorganisms. Pathogenic microorganisms are recognized through their specific molecular structures, called pathogen-related molecular patterns, by pattern recognition receptors of the host, which produce a series of signal cascades that play an antiviral role. In the course of virus propagation and evolution, viruses have evolved various adaptations to evade the cellular immune system and successfully proliferate. PCBP1 was first cloned from a human lymphocyte cDNA library in 1994 [18]. Over the next three decades, its structure and function have been well studied, and new studies are still under way. In particular, a series of studies have suggested that PCBP1 may be involved in immune responses through different pathways. PCBP1 participates in several virus-related signaling pathways, and its expression is closely related to viral replication. PCBP1 is involved in antiviral innate immunity. PCBP1 can not only participate in STAT3 (Signal transducer and activator of transcription 3) mediated inhibition of NF-κB (nuclear factor κB) activity [19, 20], but also participate in antiviral innate immune regulation through cGAS (cyclic GMP-AMP synthase) protein [21]. PCBP1 is also involved in MAVS (mitochondrial antiviral signaling) pathways for tuning antiviral immunity and preventing inflammation [22]. Therefore, we intend to conduct in-depth research on porcine PCBP1, hoping to find new directions for the prevention and control of porcine viral diseases.

The porcine kidney cells 15(PK-15) used in this study were derived from adult porcine kidney PK-2a cells established in 1955 and subsequently collected by the ATCC. Today it is one of the most commonly used porcine passageway cell lines and has been widely used in many research fields [23–25]. In this study, the porcine PCBP1 gene was cloned from PK-15 cells and bioinformatics analysis was performed to predict the structure and possible function of its protein. In addition, the subcellular location and differential expression of PCBP1 gene in various tissues were analyzed. Finally, the effects of PCBP1 on apoptosis and cell cycle were analyzed. Our results suggest that PCBP1 plays an important role in apoptosis and cell cycle regulation in PK-15 cells and possibly in pigs.

## Results

### Molecular cloning of porcine PCBP1

**Fig. 1:**
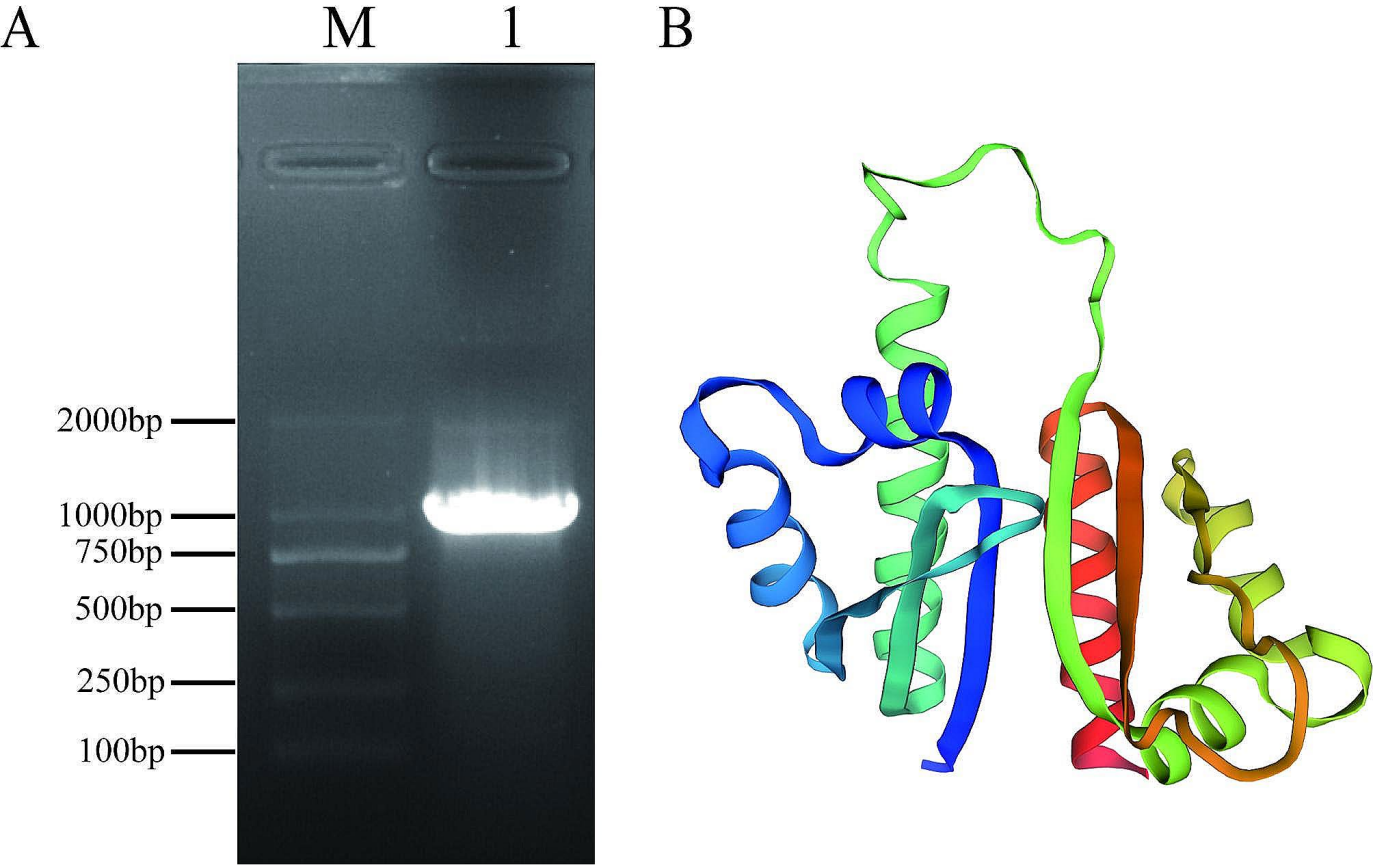
Cloning and sequencing of porcine PCBP1. (**A**) The full-length coding sequence of porcine PCBP1 gene was amplified by PCR. Lane 1, PCR products; Lane M, DNA marker. (**B**) Prediction of the 3D structure of porcine PCBP1 (based on SWISS-MODEL).

To clone porcine PCBP1, cDNA generated from PK-15 cells was used as a template for PCR, and an amplified fragment was obtained (Fig. 1A); the fragment had a clear and bright band, which was consistent with the expected result. After ligation, transformation and bacterial selection, PCR identification of the bacterial plasmids was performed. Positive colonies were verified by sequencing. The coding sequence (CDs) of PCBP1 showed 100% similarity to a previously predicted sequence (XM_003125057.4). The length of the PCBP1 CDs is 1071 bp, and the CDs encodes a protein containing 356 amino acids. The structure of PCBP1 was predicted using the SWISS-MODEL [26], and porcine PCBP1 was found to be mainly composed of α-helices and β-folds (Fig. 1B).

### Sequence analysis and phylogenetic tree construction of porcine PCBP1

**Fig. 2:**
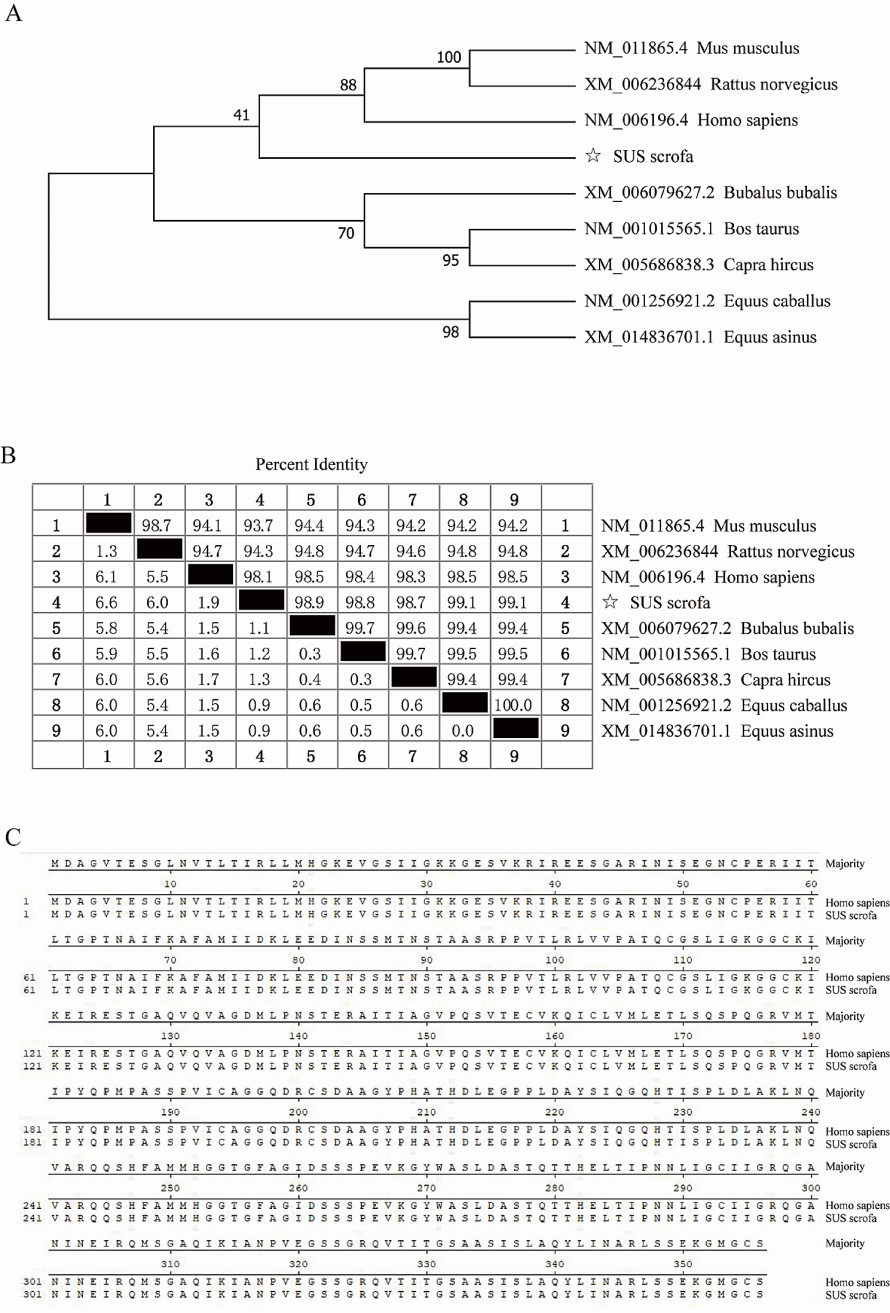
Sequence analysis of porcine PCBP1. (**A**) Phylogenetic tree analysis of PCBP1 CDs. (**B**) Homology analysis of PCBP1 in *Homo sapiens*, *Mus musculus*, *Rattus norvegicus*, *Bos taurus*, *Equus caballus*, *Capra hircus*, *Bubalus bubalis*, *Equus asinus* and *Sus scrofa*. (**C**) Comparison of PCBP1 amino acid sequences between *Sus scrofa* and *Homo sapiens*

To analyze the similarity and phylogenetic relationships of PCBP1 to its homologous proteins from other species, a multiple sequence alignment was established for these cDNA sequence with Clustal W [27], and a phylogenetic tree was constructed with MEGA 7.0 (Fig. 2A). The results of the similarity comparison showed that the PCBP1 gene was highly conserved in different species. The similarity between the sequence of porcine PCBP1 and other species was 93.7–99.1% (Fig. 2B). At the protein level, the amino acid sequence of PCBP1 between pigs and humans were identical (Fig. 2C).

### Expression abundance of PCBP1 in different tissues from 30-day-old pigs

**Fig. 3:**
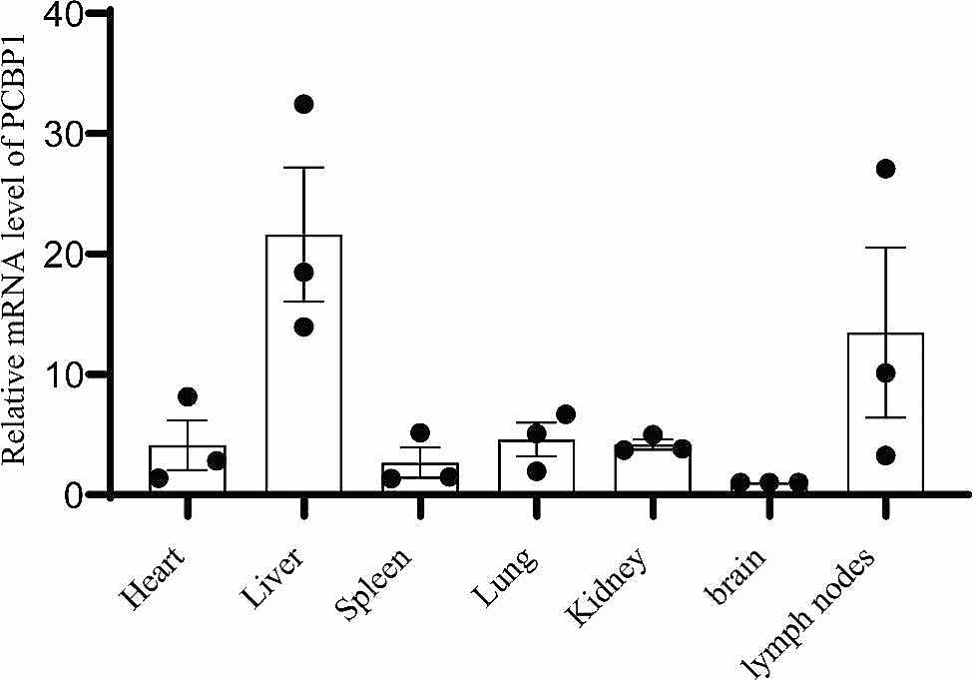
Relative expression of PCBP1 mRNA in pig tissues. RT-qPCR analysis was performed. β-actin was used as the internal control against which the PCBP1 mRNA expression was normalized

To characterize the expression level of PCBP1 mRNA in different tissues, several tissues (heart, liver, spleen, lung, kidney, brain, and lymph nodes) from 30-day-old pigs were examined by RT-qPCR. The results showed that porcine PCBP1 mRNA could be detected in all seven tissues, and the lowest expression abundance was detected in brain, while PCBP1 mRNA expression in liver and lymph nodes were significantly higher than that in other tissues (Fig. 3). This finding suggests that PCBP1 protein may play a more important role in the liver and lymph nodes than in other tissues.

### Subcellular localization of PCBP1 in PK-15 cells

**Fig. 4:**
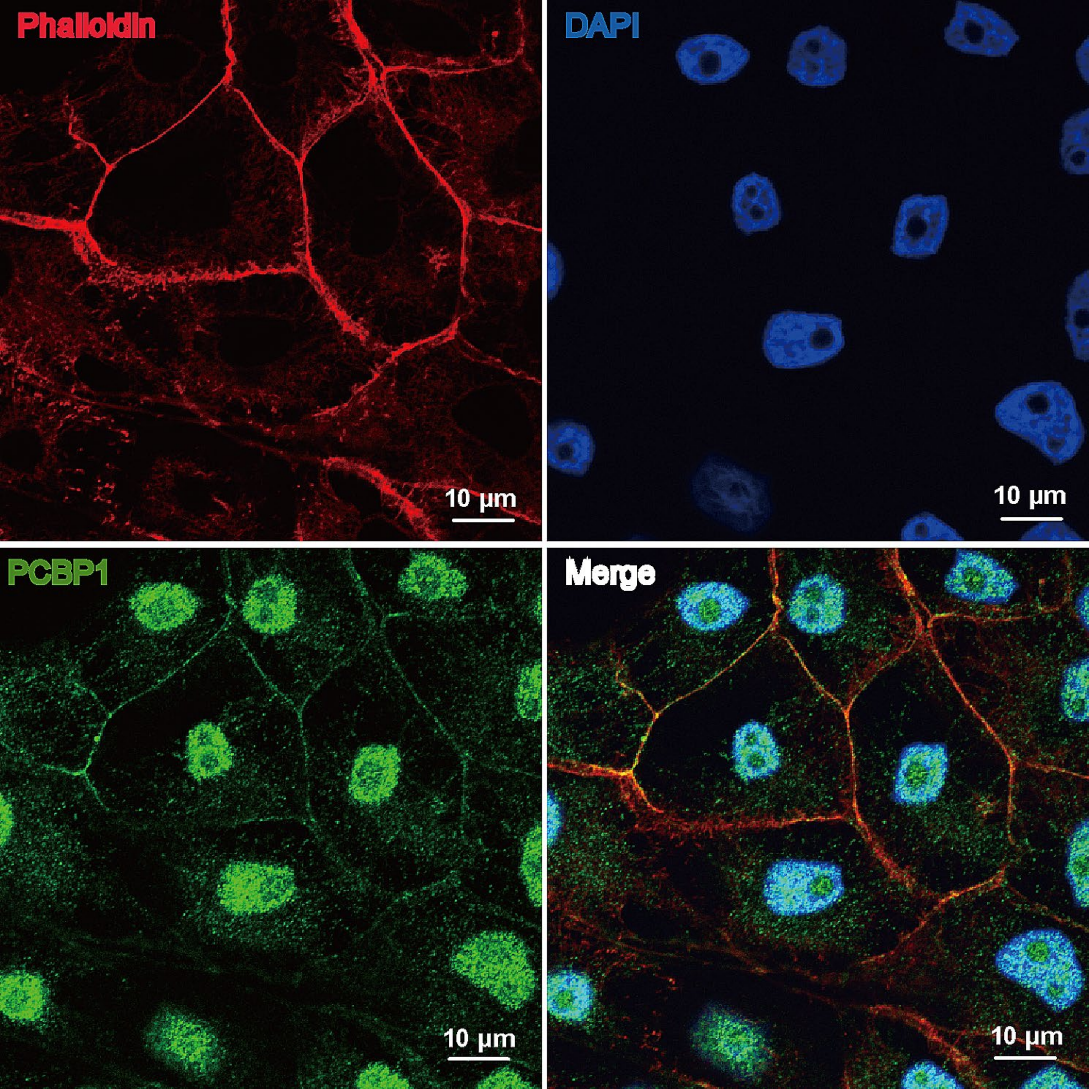
Localization of PCBP1 by immunofluorescence assay. Red fluorescence signals show samples stained with phalloidin. Blue fluorescence signals show samples stained with DAPI. Green fluorescence signals show samples stained with anti-PCBP1 antibody and FITC-conjugated goat anti-rabbit IgG. Scale bar, 10 μm

To examine the subcellular localization of porcine PCBP1, subcellular staining was performed. Immunofluorescence assay was used to detect the subcellular localization of porcine PCBP1 protein, and the results are shown in Fig. 4. Under the laser confocal microscope, the outline of PK-15 cells was clearly visible, and the cytoplasm and nucleus were clearly stained. Specifically, the PCBP1 protein was distributed in both the cytoplasm and nucleus, with more protein present in the nucleus than in the cytoplasm. In the cytoplasm, PCBP1 was scattered and concentrated on the perinuclear side and near the cell membrane. This result is consistent with the subcellular localization of human PCBP1 [28].

### Overexpression and knockdown of PCBP1 in PK-15 cells

**Fig. 5:**
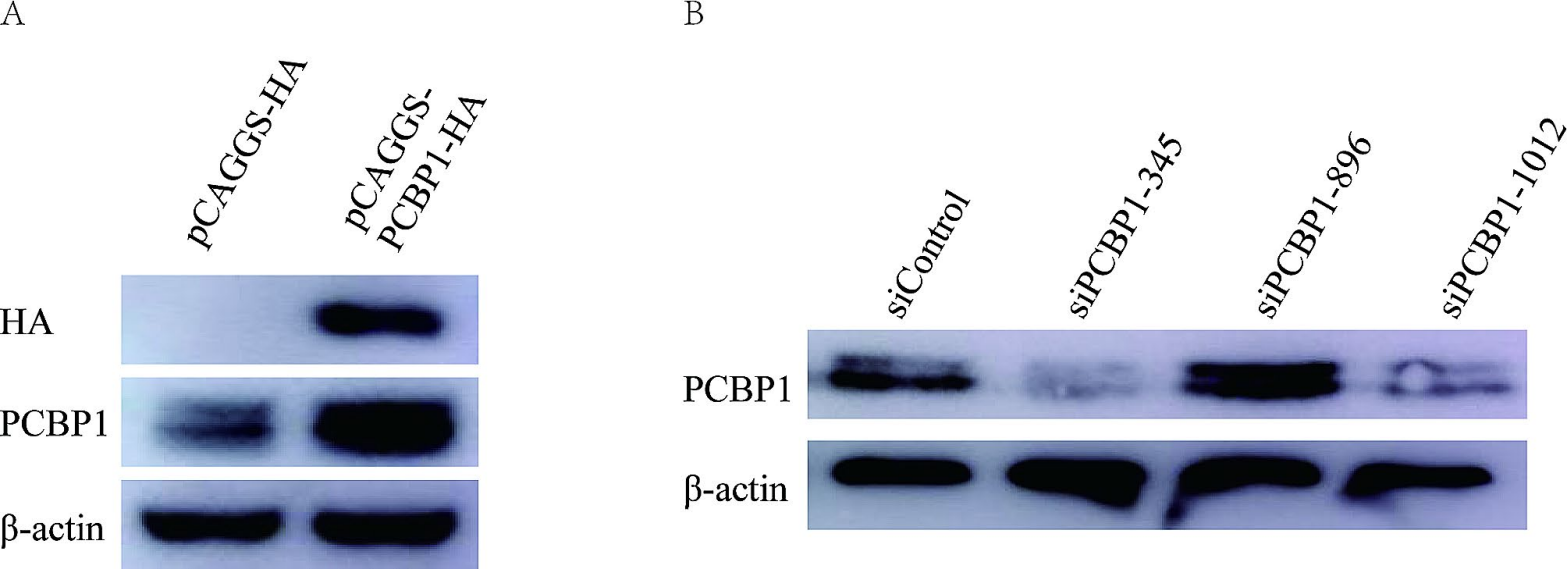
Overexpression and knockdown of PCBP1 in PK-15 cells. PK-15 cells were transfected with vector or siRNA for overexpression or knockdown of PCBP1. Expression levels of PCBP1 protein were determined by Western blotting. β-actin was used as a protein control. (**A**) The control vector (pCAGGS-HA) and expression vector (pCAGGS-HA-PCBP1) were transfected into PK-15 cells for 24 h. (**B**) Control and PCBP1 siRNAs were transfected into PK-15 cells for 24 h

In this study, the PCBP1 expression vector pCAGGS-HA -PCBP1 was successfully constructed. As confirmed by western blotting, PCBP1 was successfully expressed (Fig. 5A). Three small interfering RNAs (siRNAs ) were designed and synthesized, two of which can inhibit PCBP1 expression in PK-15 cells (Fig. 5B).

### Effect of PCBP1 on cell apoptosis

**Fig. 6:**
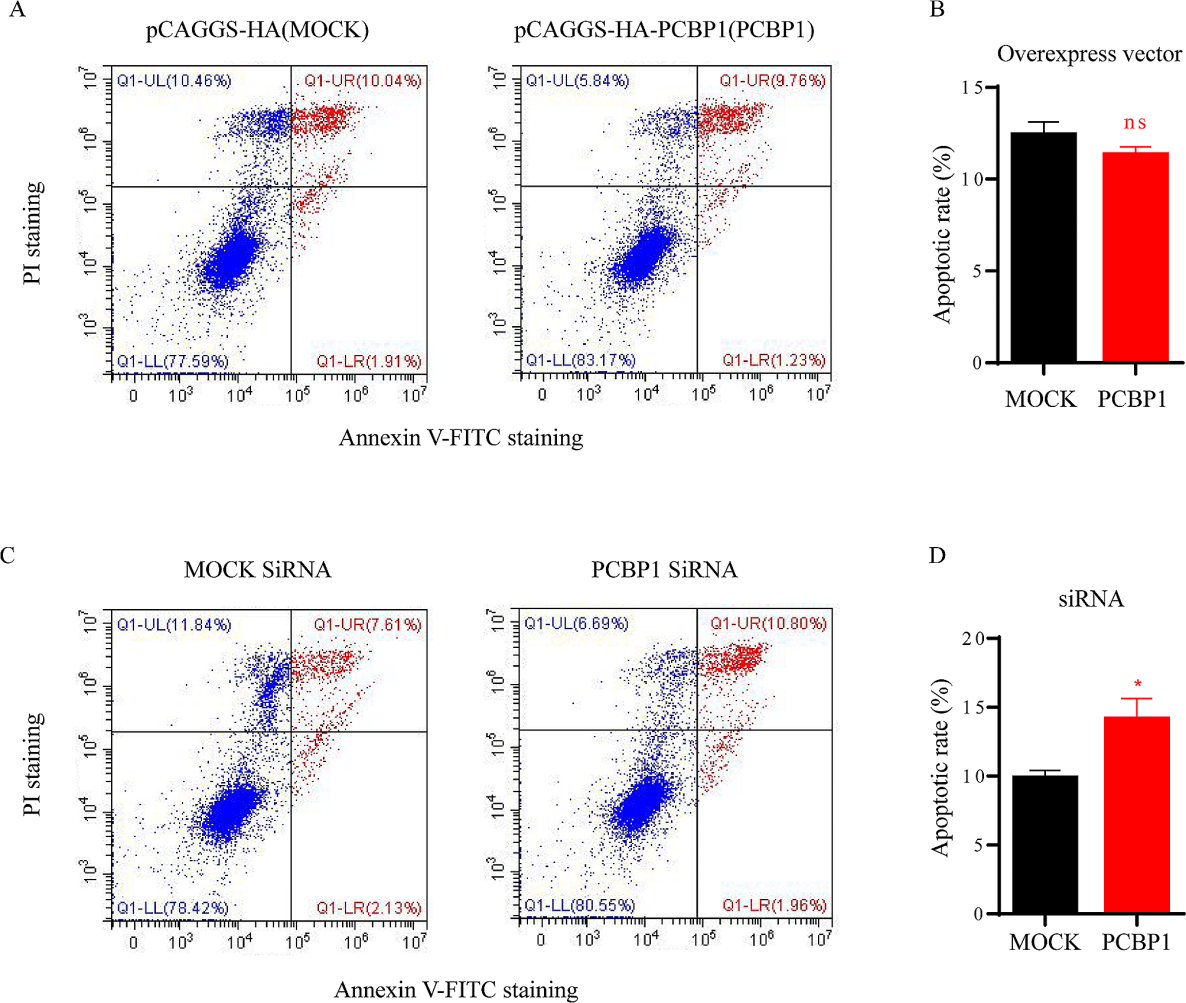
Effect of PCBP1 on apoptosis in PK-15 cells. The cells were stained with Annexin V-FITC/PI. Cell apoptosis analysis was performed by flow cytometry. A, B) pCAGGS-HA-PCBP1 and pCAGGS-HA were transfected into PK-15 cells for 24 h. The cells were stained and analyzed by flow cytometry (**A**) and presented as a percentage of apoptotic cells (**B**). (**C, D**) Mock siRNA and PCBP1 siRNAs were transfected into PK-15 cells for 24 h, the cells were stained, and the flow cytometry data (**C**) is presented as a percentage of apoptotic cells (**D**)

To detect the effect of PCBP1 on apoptosis of PK-15 cells, PCBP1 was overexpressed in PK-15 cells and apoptosis was detected by flow cytometry. PK-15 cells were transfected with pCAGGS-HA-PCBP1 overexpression plasmid and pCAGGS-HA empty plasmid. Twenty-four hours after transfection, cells were collected and detected by flow cytometry. The results showed that there was no significant difference in cell apoptosis ratio between the mock group and the PCBP1 overexpression group (Fig. 6A and B). The effect of PCBP1 knockdown on cell apoptosis in PK-15 cells was also examined. Interestingly, when PCBP1 expression was suppressed, the proportion of apoptotic cells increased, especially in the proportion of late apoptotic cells (Fig. 6C and D).

### Effect of PCBP1 on cell cycle

**Fig. 7:**
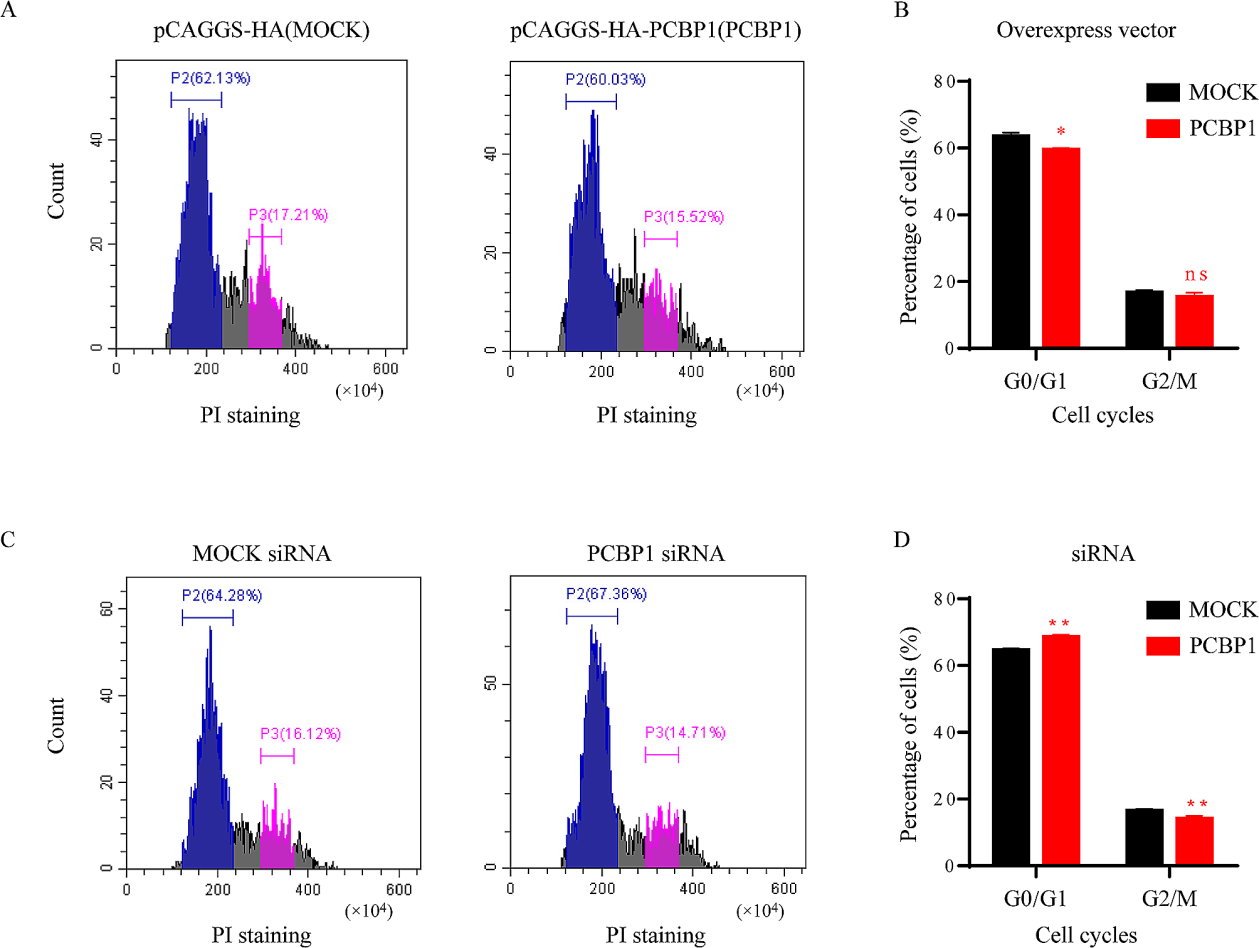
Effect of PCBP1 on cell cycle in PK-15 cells. The cells were stained with Annexin PI. Cell cycle analysis was performed by flow cytometry. (**A, B**) The plasmids pCAGGS-HA-PCBP1 and pCAGGS-HA were transfected into PK-15 cells for 24 h, and cells were stained and analyzed by flow cytometry (**A**) and presented as the percentage of G0/G1 and G2/M stages (**B**). (**C, D**) MOCK siRNA and PCBP1 siRNA were transfected into PK-15 cells for 24 h, and cells were stained and analyzed by flow cytometry (**C**) and presented as a percentage of G0/G1 and G2/M stages (**D**)

Similarly, to detect the effect of PCBP1 on cell cycle, we overexpressed and inhibited PCBP1 expression in PK-15 cells. Cell cycle analysis was performed on cells treated for 24 h. The results showed that when PCBP1 was overexpressed in PK-15 cells, the number of cells in G0/G1 phase decreased, while the number of cells in G2/M phase did not change significantly (Fig. 7A and B). When PCBP1 expression was inhibited, cells were blocked in the G0/G1 phase, and the number of cells decreased in G2/M phase (Fig. 7C and D). The results showed that when PCBP1 protein expression decreased, cells were arrested in the G0/G1 phase.

## Discussion

In this study, the CDs of PCBP1 gene in PK-15 cells was successfully cloned, which was 1071 bp, could encode 356 amino acids and was consistent with the predicted sequence (XM_003125057.4) in NCBI (national center for biotechnology information). The CDs of porcine PCBP1 was compared with several other species, and the results showed that PCBP1 was highly conserved in animals. These results indicated that PCBP1 may be an important protein that is highly conserved during evolution.

Subcellular localization showed that PCBP1 protein was present in both the cytoplasm and nucleus, consistent with the reported distribution of human PCBP1 [28]. In PK-15 cells, PCBP1 expression is mainly located in the nucleus, which is consistent with the localization of human PCBP1 protein. In addition, PCBP1 in the cytoplasm was mainly located on the perinuclear side and near the cell membrane, suggesting that PCBP1 may association with membrane structures. In mouse, depletion of PCBP1 induced mitochondrial dysfunction, demonstrated by reduction of Mitofusin 2 and ATP levels in liver [29]. PCBP1 regulated Enterovirus 71 replication in the host specialized membrane-associated replication complex [30].

We selected 30-day-old pigs, dissected several different tissues, and tested the expression level of PCBP1 mRNA. The results showed that PCBP1 had the lowest expression level in the brain, and relatively high in the liver and lymph nodes. It is well known that the liver’s functions include metabolism, detoxification, and hematopoiesis; especially in young animals, the liver is an important hematopoiesis organ [31]. It has also been reported that PCBP1 is associated with hematopoietic function [32]. As an important immune organ, lymph nodes play a critical role in the immune system. The high expression of PCBP1 in lymph nodes suggests that PCBP1 may have some immune function. There are reports that PCBP1 is a novel mediator of antiviral innate immunity and has been shown to increase replication of Classical swine fever virus [33]. Furthermore, PCBP1 has recently been shown to reduce IFN induction by degradation of MAVS [22]. Moreover, ChenYang Liao, CaoQi Lei and HongBing Shu confirmed that PCBP1 plays an important role in cGAS-mediated innate immune response to DNA virus infection by promoting cGAS binding to viral DNA [21]. These results suggest that PCBP1 is an important multi-functional protein, involved in a variety of life processes.

Finally, we investigated the effect of PCBP1 on apoptosis and cell cycle. In terms of cell apoptosis, PCBP1 overexpression did not affect cell apoptosis rate, but it was significantly increased when PCBP1 was inhibited. PCBP1 has been reported to bind heavily oxidized RNA, inhibiting apoptosis under oxidative conditions [34]. Moreover, long noncoding RNA SNHG1 (small nucleolar RNA host gene 1) inhibits apoptosis by up regulating GNAI2 (G protein alpha inhibiting activity polypeptide 2) and PCBP1 [35]. Under starvation conditions, PCBP1 promoted apoptosis of tumor cells through the autophagy pathway [17]. However, there are also reports that PCBP1 overexpression elicits cycle arrest, apoptosis induction, and p73 splicing in human cervical carcinoma cells [36]. In most cases, viral infection induces apoptosis [37–39], but although the mechanism is unclear, PCBP1 may be a potential target for initial investigation.PCBP1 overexpression had little effect on cell cycle in PK-15 cells. However, PK-15 cells were arrested in G0/G1 phase when PCBP1 expression was inhibited. A comprehensive analysis of the effects of PCBP1 on cell cycle and apoptosis showed that the lack of PCBP1 would affect the normal physiological function of cells. Moreover, previous investigations have shown consistent results [15, 36], similar to the current study on the effect of human PCBP1 on the cell cycle. This result indicates that PCBP1 is an important cellular protein in the cell cycle and deserves further study.

## Conclusion

In this study, we cloned porcine PCBP1, studied its subcellular localization and tissue expression level in different tissues, and characterized its effect on cell cycle and apoptosis. This study provides a basis for further research on the functions of porcine PCBP1.

### Methods

#### Strains, plasmids, and antibodies

PK-15 cells were cultured in Dulbecco’s Modified Eagle’s Medium (DMEM, Gibco, USA) supplemented with 10% fetal bovine serum (FBS, Gibco, USA) at 37 °C in a humidified atmosphere containing 5% CO_2_. *Escherichia* coli TOP10 (Invitrogen, Beijing, China) was used for plasmid amplification and was grown in LB medium at 37 °C. The plasmid pMD 18-T vector (Invitrogen, Beijing, China) was used to clone the PCBP1 gene and the plasmid pCAGGS-HA was used to produce HA-tagged PCBP1 proteins. Anti-PCBP1, anti-HA and anti-β-Actin antibodies were purchased from Proteintech (Wuhan, China).

### Cloning and sequencing of porcine PCBP1

Total RNA was isolated from PK-15 cells using TRIzol reagent (Invitrogen, Beijing, China). The cDNA was generated using a HiScriptII 1st Strand cDNA Synthesis Kit (Vazyme, Nanjing, China) according to the manufacturer’s instructions. PCBP1 CDs was amplified using the following primers designed based on the previously predicted PCBP1 cDNA sequence (Accession Number: XM_003125057.4). The forward primer was 5’-ATGGATGCCGGTGTGACTG-3’ and the reverse primer was 5’-CTGCACCCCATGCCCTTC-3’. After the amplified DNA fragment was purified using the Gel Extraction Kit (TransGen, Beijing, China), it was ligated into the pMD18-T vector and transformed into the *E. coli* TOP10 strain. Positive colonies were verified by sequencing.

### Similarity comparison and phylogenetic tree construction

SWISS-MODEL (https://swissmodel.expasy.org) was used for PCBP1 homology modeling. PCBP1 gene sequences in various species, including *Homo sapiens* (NM_006196.4), *Mus musculus* (NM_011865.4), *Rattus norvegicus* (XM_006236844), *Bos Taurus* (NM_001015565.1), *Equus caballus* (NM_001256921.2), *Capra hircus* (predicted, XM_005686838.3), *Bubalus bubalis* (predicted, XM_006079627.2), and *Equus asinus* (XM_014836701.1), were downloaded from NCBI (https://www.ncbi.nlm.nih.gov/), followed by amino acid multiple sequence alignment with DNAStar. The phylogenetic tree was constructed using the neighbor-joining (NJ) method in MEGA 7.0 software, and 1 000 self-spreading analyses were performed.

### Tissue specific expression analysis

Based on the sequencing results, primers for PCBP1 qPCR detection were selected. The forward primer was 5’-CAGTCTGTCACCGAGTGTGT-3’ and the reverse primer was 5’-GTCATGACTCTCCCTTGCGG-3’. The relative mRNA expression of PCBP1 in pig tissues (heart, liver, spleen, lung, kidney, brain, and lymph nodes) was detected by RT-qPCR. The tissues were derived from healthy 30-day-old piglets (2 females and 1 male) purchased from a commercial pig farm (Xiongfeng Sige, Zhengzhou, China). No obvious clinical signs or significant temperature changes were observed in these piglets. Animal experiments comply with the ethical standards of animal experiments and the relevant provisions of animal welfare. Briefly, piglets were anaesthetized with 30 g/L sodium barbiturate, and euthanized through i.v. application of 0.5mL/kg of 10% KCl via the ear vein. After euthanized, piglets were dissected for tissue collection. The tissues were ground on ice and total RNA was extracted from each tissue and complementary DNA was synthesized. RT-qPCR was performed using Power SYBR Green PCR Master Mix (Vazyme, Nanjing, China). Each sample was triplicated. β-actin was used as the internal control. The relative expression levels of PCBP1 in different tissues were analyzed by -2^△△T^. Data are expressed as the mean ± SEM (standard deviation).

### IFA assay

PK-15 cells seeded on coverslips were fixed with 4% paraformaldehyde (Servicebio, Wuhan, China) for 10 min at 4 °C, blocked with 3% BSA for 30 min at room temperature and then incubated with primary antibodies against PCBP1 (Proteintech, Wuhan, China) at 4 °C overnight. The secondary antibody used was FITC-conjugated anti-rabbit IgG (Proteintech, Wuhan, China). The cells were treated with phalloidin (Servicebio, Wuhan, China) which labeled cytoskeleton. In addition, the cells were stained with DAPI (Servicebio, Wuhan, China) to visualize the nuclei. Images were obtained by fluorescence microscope (Zeiss, Oberkochen, Germany).

### Overexpression of PCBP1 in PK-15 cells

The verified pMD18-T-PCBP1 was used as a template to amplify the cDNA fragment encoding the mature PCBP1 protein (without the signal peptide) by PCR and then ligated into the pCAGGS-HA vector. The pCAGGS-HA-PCBP1 plasmid was transfected into PK-15 cells by ExFect®2000 Transfection Reagent (Vazyme, Nanjing, China) according to the manufacturer’s instructions. Cell samples were collected 24 h after transfection for Western blot analysis.

### RNA interference of PCBP1 expression

Three small interfering RNA sequences targeting porcine PCBP1 (siRNA345, siRNA894 and siRNA1012) and one irrelevant interference sequence were designed separately according to PCBP1 sequence. The sequences of these siRNAs were siRNA345 (5’-GCGGCUGUAAGAUCAAAGATT-3’), siRNA894 (5’-GCGCCAACAUUAAUGAGAUTT-3’) and siRNA1012 (5’-GGCCCAAUAUCUAAUCAAUTT-3’). These sequences were synthesized by GenePharma Company. PK-15 cells were transfected with siRNA using ExFect®2000 Transfection Reagent and cell samples were collected 24 h after transfection for Western blot analysis.

### Western blotting

Western blotting was used to identify PCBP1 expression. Total protein was isolated by adding lysis buffer (Beyotime, Shanghai, China) to the cells. Cell extracts were separated by SDS-PAGE and electro blotted onto a polyvinylidene difluoride (PVDF) membrane (Millipore, Billerica, USA). Then, the membranes were incubated with specific primary antibodies, followed by incubation with appropriate horseradish peroxidase (HRP)-conjugated secondary antibodies. Signals were detected using an enhanced chemiluminescence detection kit (Millipore, Billerica, USA).

### Cell apoptosis analysis

Annexin V/PI double staining was used to detect phosphatidylserine valgus to evaluate apoptosis. The cells were stained with an Annexin V-FITC/PI Apoptosis Detection Kit (Solarbio, Beijing, China) and apoptosis was detected by flow cytometry. Cell apoptosis data were analyzed using CytExpert software (Beckman Coulter, USA). All experiments were performed in triplicate.

### Cell cycle analysis

When PK-15 cells reached 60% confluence in the well, the plasmids pCAGGS-HA-PCBP1 and pCAGGS-HA were transfected for 24 h. In another group, non-targeting siRNA and PCBP1 siRNA were also transfected into PK-15 cells for 24 h. The cells were then harvested, washed twice with ice-cold PBS, and fixed with 95% ethanol overnight. Afterward, cells were stained with 50 µg/ml propidium iodide (PI) at 37 °C in the dark for 30 min. Then, the cells were analyzed by flow cytometry.

### Statistical analysis

Statistical analysis was performed using GraphPad Prism 6 (GraphPad Software). Differences between groups were evaluated for significance by one-way analysis of variance with Dunnett’s post-comparison test for multiple groups. All experimental groups were compared to the control group. * indicates *p* < 0.05; ** indicates *p* < 0.01; and *** indicates *p* < 0.001. Experimental data are presented as mean ± SEM, and differences with *p* values < 0.05 were considered statistically significant.

### Electronic supplementary material

Below is the link to the electronic supplementary material.

Supplementary Material 1

## Data Availability

The datasets used and/or analyzed during the current study are available from the corresponding author on reasonable request. DNA sequences have been deposited in GenBank (accession numbers: OR037304).

## Abbreviations

PCBP1: Poly C Binding Protein 1
IFA: Immunofluorescence assay
RT-qPCR: Reverse transcription-quantitative PCR
PK-15: Porcine kidney cells 15
LC3 B: Microtubule-associated protein light chain 3
STAT3: Signal transducer and activator of transcription 3
NF-κB: Nuclear factor κB
cGAS: Cyclic GMP-AMP synthase
MAVS: Mitochondrial antiviral signaling
CDs: Coding sequence
siRNA: Small interfering RNA
NCBI: National center for biotechnology information
SNHG1: Small nucleolar RNA host gene 1
DMEM: Dulbecco’s modified Eagle’s medium
FBS: Fetal bovine serum
PVDF: Polyvinylidene difluoride
PI: Propidium iodide
