# Supplementary material for: Molecular and functional characterization of porcine poly C binding protein 1 (PCBP1)
Source: BMC Vet Res. 2024 Jan 13;20:25. doi: 10.1186/s12917-023-03861-4 (PMC10787444; doi:10.1186/s12917-023-03861-4)
Supplement: Supplementary file 1 — Supplementary Material 1 [file 12917_2023_3861_MOESM1_ESM.pdf]

All

Fig1A

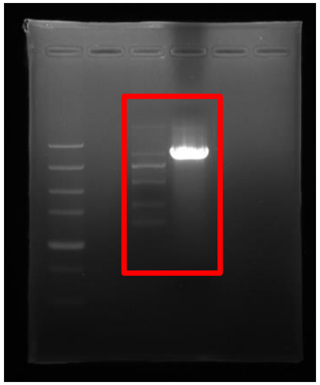

Fig5A
Actin

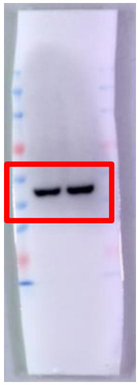

Fig5A
HA

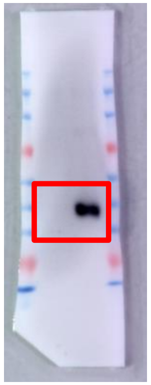

Fig5A
PCBP1

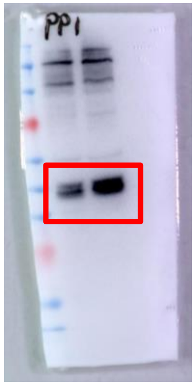

Fig5B Actin

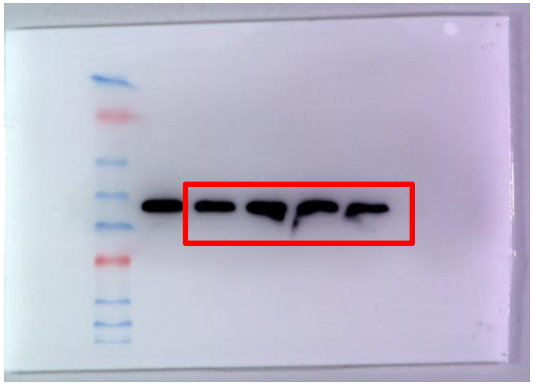

Fig5B PCBP1

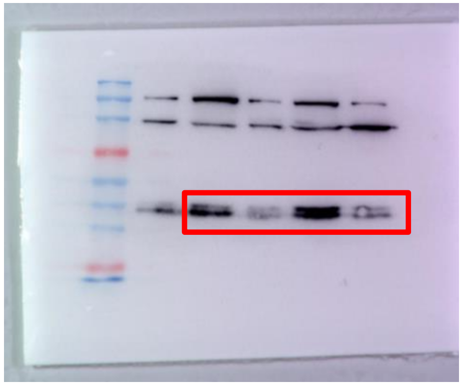

Fig1A

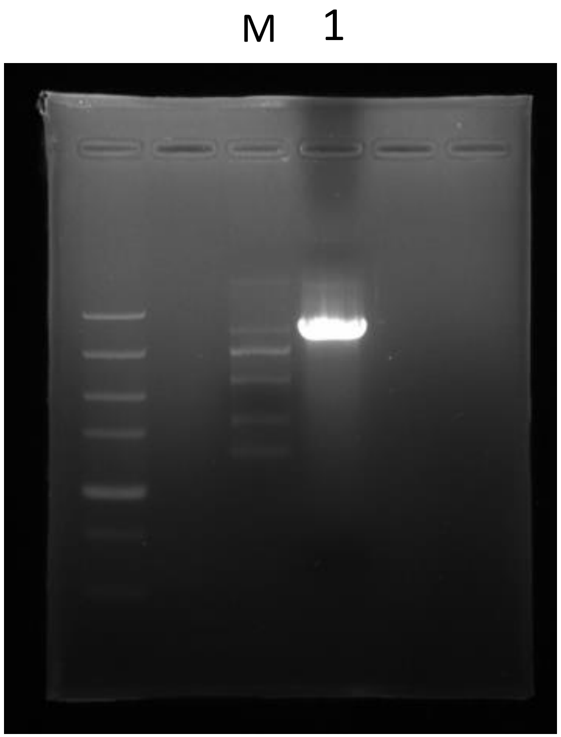

Fig1A. The full-length coding sequence of the porcine PCBP1 gene was amplified by PCR. Lane 1, PCR products; Lane M, DNA marker.

Fig5A

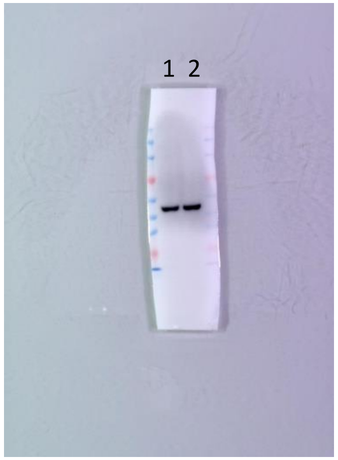

Fig5A
Actin

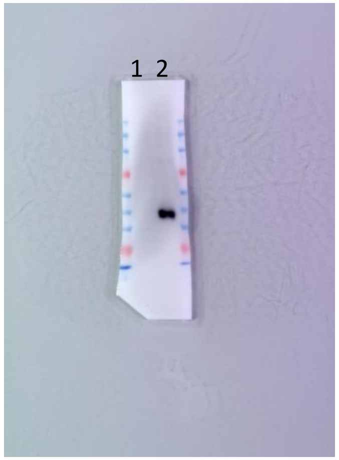

Fig5A
HA

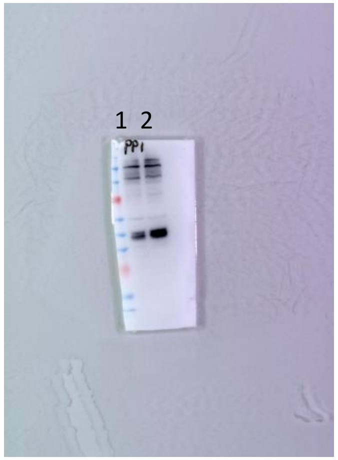

Fig5A
PCBP1

1. Transfected with control vector (pCAGGS-HA)
2. Transfected with PCBP1 expression vector (pCAGGS-HA-PCBP1)

Fig5B

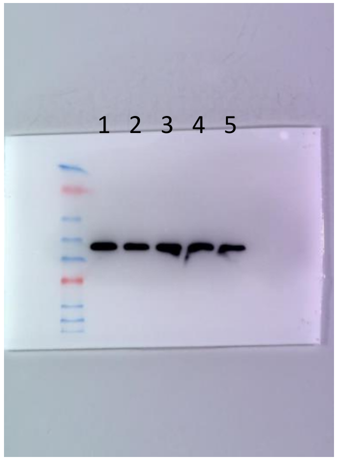

Fig5B Actin

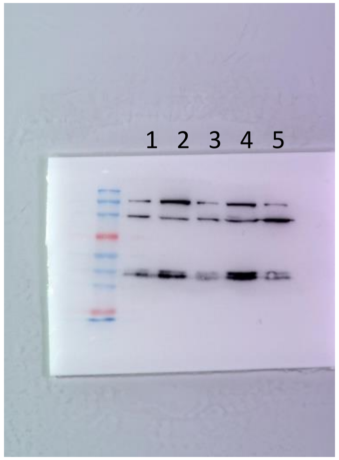

Fig5B PCBP1

1. Other
2. siControl
3. siPCBP1-345
4. siPCBP1-896
5. siPCBP1-1012
